# The Prevalence and Burden of Avoidant/Restrictive Food Intake Disorder (ARFID) Symptoms in the Adult General Population of the UK and USA

**DOI:** 10.1002/eat.24588

**Published:** 2025-10-29

**Authors:** Grace Brownlow, Rosie Flack, Helen Burton‐Murray, Olafur Palsson, Imran Aziz

**Affiliations:** ^1^ Division of Clinical Medicine School of Medicine and Population Health, University of Sheffield Sheffield UK; ^2^ Academic Department of Gastroenterology Sheffield Teaching Hospitals Sheffield UK; ^3^ Department of Medicine, Division of Gastroenterology Massachusetts General Hospital Boston Massachusetts USA; ^4^ Harvard Medical School Boston Massachusetts USA; ^5^ Center for Functional GI and Motility Disorders University of North Carolina at Chapel Hill Chapel Hill North Carolina USA

**Keywords:** ARFID, multimorbidity, NIAS

## Abstract

**Introduction:**

Avoidant/Restrictive Food Intake Disorder (ARFID) is a feeding and eating disorder characterized by avoidant/restrictive eating behaviors that lead to medical and/or functional impairments. While ARFID is increasingly recognized within pediatric populations and specialist clinics, data on its prevalence and burden within the adult general population remain sparse. We sought to address this knowledge gap.

**Methods:**

We conducted a population‐based internet survey with predefined demographic quotas across the UK and USA. The survey included the Nine‐Item ARFID Screen (NIAS), the Rome IV diagnostic questionnaire for disorders of gut‐brain interaction (e.g., irritable bowel syndrome, functional dyspepsia), and questions regarding demographics, body mass index, somatic symptoms, anxiety and depression, quality of life, and healthcare utilization.

**Results:**

Among 4002 participants (mean age 47.1 years, 50% female), 26.0% screened positive for ARFID. Prevalence was significantly higher in females versus males (29.6% vs. 22.1%, OR 1.48, 95% CI 1.28–1.71) and varied by age: 18–39 years (31.6%), 40–64 years (25.0%), and ≥ 65 years (16.1%; *p* < 0.001). Participants with a positive ARFID screen demonstrated significantly higher rates of underweight status, disorders of gut‐brain interaction, mood disturbances, somatic symptoms, reduced mental and physical quality of life, and increased healthcare utilization compared to unaffected individuals. Increasing severity of ARFID correlated with greater general health impairment (all *p* < 0.001).

**Conclusion:**

A positive ARFID screen is common within the adult general population, affecting 1‐in‐4 people, and associated with a substantial health burden. Increased awareness of ARFID will facilitate clinical service provision and guide future research.

**Summary**

- This general population‐based survey of 4002 adults found that one in four adults screened positive for ARFID, with a significantly higher prevalence in females and younger adults.
- Those with a positive ARFID screen experienced greater multi‐morbidity compared to unaffected individuals.
- Increased clinical awareness and multidisciplinary care approaches are needed for individuals with ARFID.

## 1 Introduction

Avoidant/Restrictive Food Intake Disorder (ARFID) was introduced into the DSM‐5 in 2013 (American Psychiatric Association 2022). It is defined by persistent failure to meet nutritional and/or energy needs, resulting in significant weight loss, nutritional deficiencies, dependence on enteral feeding or oral supplements, and/or substantial psychosocial impairment (American Psychiatric Association 2022). Unlike anorexia nervosa and bulimia nervosa, ARFID does not stem from concerns about body weight or shape. Instead, restrictive eating in ARFID is associated with at least one of the following three domains: (i) lack of interest in eating (i.e., poor appetite), (ii) sensory‐based avoidance (i.e., picky eating due to issues with taste, texture, smell), and/or (iii) fear of aversive consequences (e.g., choking, vomiting, or abdominal pain after eating). When medical conditions are present, avoidant/restrictive eating must exceed what would be expected and warrant independent clinical attention to meet ARFID criteria (American Psychiatric Association 2022).

The prevalence of ARFID is incompletely understood. A recent systematic review and meta‐analysis, comprising 122,861 individuals across 26 studies, indicated a pooled prevalence of ARFID as 4.51% or 11.14%, depending on the analytical model used (Nicholls‐Clow et al. 2024). The prevalence ranged from 0.8% to 28% in non‐clinical samples, and from 0.8% to 64% in clinical samples (Nicholls‐Clow et al. 2024). However, significant methodological heterogeneity was observed between studies, including differences in study design (e.g., retrospective chart reviews vs. cross‐sectional studies etc.), sample sizes, population settings (e.g., clinical vs. non‐clinical), age ranges, and questionnaires used to screen for or diagnose ARFID (Nicholls‐Clow et al. 2024). Most studies were also conducted in pediatric and adolescent populations. The investigators concluded that large‐scale studies focusing on specific sample populations (e.g., adults) could enhance the accuracy and utility of prevalence estimates (Nicholls‐Clow et al. 2024). Furthermore, ARFID has been associated with multi‐morbidity, including mood disturbances, gastrointestinal symptoms (e.g., irritable bowel syndrome), and somatic symptom reporting, although data in this area remain limited (Staller et al. 2023; Mikhael‐Moussa et al. 2025).

To address these gaps, we aimed to investigate the prevalence and burden of ARFID symptoms within the general adult population. Our exploratory hypotheses were that: (i) ARFID symptoms are common in this group, (ii) those with positive ARFID screens experience an increased health burden compared to those without ARFID, and (iii) with increasing severity of ARFID, the higher the general health burden (Mikhael‐Moussa et al. 2025).

## 2 Methods

### 2.1 Study Design and Participant Recruitment

Between October 31 and December 15, 2023, we administered an online survey to an adult general population from the UK and USA using the Qualtrics Inc. platform (Provo, Utah, USA). To achieve demographically representative samples based on age and gender, we implemented predetermined quota targets for both nations. These quotas ensured an age distribution of 40% participants aged 18–39, 40% aged 40–64, and 20% aged 65+, while maintaining equal gender representation (50% male, 50% female). Consistent with our previous population‐based studies (Sperber et al. 2021), we aimed for a sample size of approximately 2000 adults per country.

Participants were invited to complete an online “general health survey” without specific reference to eating disorders. No personally identifiable information was collected. Quality assurance procedures included restricting responses to one per device, requiring mandatory completion of applicable questions, and excluding participants who failed two attention checks or displayed excessive inconsistency across repeated diagnostic questions.

### 2.2 Questionnaires

The survey consisted of a series of questionnaires. It collected data on demographics, body mass index (BMI), past medical history and use of healthcare services. The Nine‐Item ARFID Screen (NIAS) was used to assess for ARFID symptoms and the Rome IV Diagnostic Questionnaire screened for DGBI (e.g., irritable bowel syndrome, functional dyspepsia). Patient health questionnaires measured anxiety, depression and somatic symptoms. Physical and mental quality of life scores were obtained through a global health questionnaire. Further details are provided below:

#### 2.2.1 Medical History and Healthcare Utilization

The participant was asked if they had ever been diagnosed with diabetes, celiac disease, inflammatory bowel disease, GI cancer, migraines, or fibromyalgia. An abdominal surgical history was also undertaken. Participants were asked to indicate if they had ever had surgery where the gallbladder, appendix, uterus or part of the intestine, had been removed.

To investigate medication use, a list of nine medications that are often used in the management of GI and mood disorders was provided. The respondent had to indicate if they took any of the following medications weekly: laxatives, anti‐diarrheals, antiemetics, acid suppressants, analgesics, antispasmodics, anxiolytics, antidepressants, or sedatives.

#### 2.2.2 Nine‐Item ARFID Screen (NIAS)

This is a brief screening instrument designed and validated to assess individuals for the three predominant ARFID presentations (Zickgraf and Ellis 2018). The NIAS has nine items: (1) “I am a picky eater,” (2) “I dislike most of the foods that other people eat,” (3) “The list of foods that I like and will eat is shorter than the list of foods I won't eat,” (4) “I am not very interested in eating; I seem to have a smaller appetite than other people,” (5) “I have to push myself to eat regular meals throughout the day, or to eat a large enough amount of food at meals,” (6) “Even when I am eating a food I really like, it is hard for me to eat a large enough volume at meals,” (7) “I avoid or put off eating because I am afraid of gastrointestinal discomfort, choking or vomiting,” (8) “I restrict myself to certain foods because I am afraid that other foods will cause gastrointestinal discomfort, choking or vomiting,” and (9) “I eat small portions because I am afraid of gastrointestinal discomfort, choking or vomiting” (Zickgraf and Ellis 2018).

These nine items are evenly divided into three subscales which correspond to the symptoms of one of the three domains of ARFID: Items 1–3 assess for picky eating (NIAS‐Picky), 4–6 assess for low appetite (NIAS‐Interest), and 7–9 for fear of aversive consequences (NIAS‐Fear) (Zickgraf and Ellis 2018). Respondents rate how much each statement applies to them on a scale from 0 (strongly disagree) to 5 (strongly agree). Total subscale score ranges from 0 to 15, with higher scores indicating greater severity of symptoms within that domain (Zickgraf and Ellis 2018).

Burton‐Murray et al. validated cut‐off scores for each NIAS subscale in a clinical sample: scores of ≥ 10 on the NIAS‐Picky and NIAS‐Fear subscales, and ≥ 9 on the NIAS‐Interest subscale, demonstrated good sensitivity and specificity for identifying ARFID presentation (Burton Murray et al. 2021). Therefore, we chose to apply these cut‐off scores in our study. Internal consistency in our sample was high for all subscales as indicated by Cronbach's alpha: 0.825 for NIAS‐Picky, 0.844 for NIAS‐Appetite, and 0.899 for NIAS‐Fear.

#### 2.2.3 Rome IV Diagnostic Questionnaire

This validated questionnaire is benchmarked as the diagnostic tool for disorders of gut‐brain interaction (DGBI), their inclusion into clinical trials, and for performing epidemiological surveys (Palsson et al. 2016). We categorized DGBI in accordance with the six‐region division of these disorders as per the Rome diagnostic system, i.e., esophageal, gastroduodenal, gallbladder, bowel, anorectal, and centrally mediated disorders of GI pain. However, due to a lack of cases, we excluded centrally mediated disorders of GI pain (*n* = 1) and biliary disorders (*n* = 7) from further analysis. We also focused on the two most widely recognized DGBI, that is irritable bowel syndrome (IBS, emanating from the bowel domain) and functional dyspepsia (emanating from the gastroduodenal domain).

#### 2.2.4 PHQ‐4 Anxiety and Depression

This is a valid self‐report tool that combines the Patient Health Questionnaire‐2 (PHQ‐2) and the General Anxiety Disorder‐2 (GAD‐2) to screen for anxiety and depression symptoms (Kroenke et al. 2009). In the PHQ‐4, respondents rate how often they have been bothered by each of the four symptoms over the last 2 weeks from not at all (0 points), to several days (1 point), to more than half the days (2 points), to nearly every day (3 points) (Kroenke et al. 2009). The symptoms questioned are: (1) Feeling anxious, nervous or on edge, (2) Not being able to stop or control worrying, (3) Little interest or pleasure in doing things, and (4) Feeling down, depressed, or hopeless. The results can be interpreted as follows: a score of 0–2 is normal, 3–5 is mild, 6–8 is moderate, and 9–12 is severe (Kroenke et al. 2009). In this study, the PHQ‐4 anxiety and depression subscales were analyzed separately, with a score of ≥ 3 used as the threshold for identifying anxiety and depression on the respective subscales.

#### 2.2.5 PHQ‐15 Somatic Symptoms

This is a validated self‐administered tool used to assess the burden of somatic symptoms (Kroenke et al. 2002, 2010). The questionnaire investigates fifteen somatic symptoms: (1) Stomach pain, (2) Back pain, (3) Pain in their arms, legs, or joints, (4) Menstrual cramps or problems with your period (for individuals who menstruate), (5) Headaches, (6) Dizziness, (7) Feeling your heart pound or race, (8) Shortness of breath, (9) Pain or problems during sexual intercourse, (10) Constipation, loose bowels, or diarrhea, (11) Nausea, gas or indigestion, (12) Feeling tired or having low energy, (13) Trouble sleeping, (14) Chest pain, and (15) Fainting spells (Kroenke et al. 2002).

Participants are asked to rate how much each symptom has bothered them over the past 4 weeks using the following three options: Not bothered at all (0 points), bothered a little (1 point), and bothered a lot (2 points) (Kroenke et al. 2002). Total scores range from 0 to 30 and can be categorized as the following: 0–4 indicates minimal somatization, 5–9 low somatization, 10–14 medium somatization, and 15–30 high somatization (Kroenke et al. 2002). In this study, the PHQ‐15 scores were dichotomized into minimal‐to‐low (PHQ‐15 < 10) and medium‐to‐high (PHQ‐15 ≥ 10) somatization. We also report the prevalence of how often each of the somatic symptoms bothered individuals “a lot”.

#### 2.2.6 PROMIS Global‐10 Quality of Life (QOL)

This is a 10‐item questionnaire designed to assess physical, mental, and social aspects of health (Hays et al. 2009). Most of the items are rated on a 5‐point Likert scale (with higher scores indicating better health). Four items in the questionnaire contribute to an overall physical health score (covering physical health, physical functioning, pain, and fatigue), while another four contribute to a mental health summary score (assessing overall quality of life, mental health, satisfaction with social activities and relationships, and emotional problems) (Hays et al. 2009). As detailed in the global health scoring manual, the raw physical and mental quality of life scores are standardized into T‐scores (PROMIS Global Health 2025). Calibration testing was carried out in a large sample of the general US population, and this established a mean T‐score of 50 and a standard deviation of 10. Therefore, a T score above 50 indicates that the participant reports a higher quality of life, while a score below 50 indicates a lower quality of life. In this study, the mean T scores for the overall mental and physical domains were calculated and used in comparisons. T scores were also categorized into below average (T < 50) and equal to or above average (T ≥ 50).

### 2.3 Statistical Analyses

All statistical analyses were conducted using SPSS version 29.0 (SPSS Inc., Chicago, Illinois, USA), with a significance level set at *p* < 0.05.

Categorical variables were displayed as total frequencies and percentages, with associations between categorical variables tested using Pearson's chi‐squared test. Odds ratios (OR) were also calculated and recorded with 95% confidence intervals (95% CI). Binary logistic regression was used to analyze categorical variables while adjusting for age and sex, given that they are potential confounders in ARFID.

For continuous data, histograms were used to assess for normal distribution. If the data was normally distributed, then the mean and standard deviation (SD) were calculated and used to summarize the variable. For parametric data, Levene's test was performed to check that groups had approximately equal variances. If this assumption was met, an independent *t*‐test was used to compare two groups, For non‐normally distributed data, the median and inter‐quartile range (IQR) were reported, with Mann Whitney U‐tests used to compare two groups. Correlations between continuous variables were assessed using Spearman's rho.

## 3 Results

### 3.1 Baseline Characteristics

A total of 4002 participants (2000 from the UK and 2002 from the USA) completed the survey. The sample was 50.0% female, with a mean of 47.1 years (SD 17.0) and 81.7% of White ethnicity. See the Table S1 for demographics across the UK and USA.

### 3.2 Prevalence of ARFID Symptoms

**FIGURE 1:**
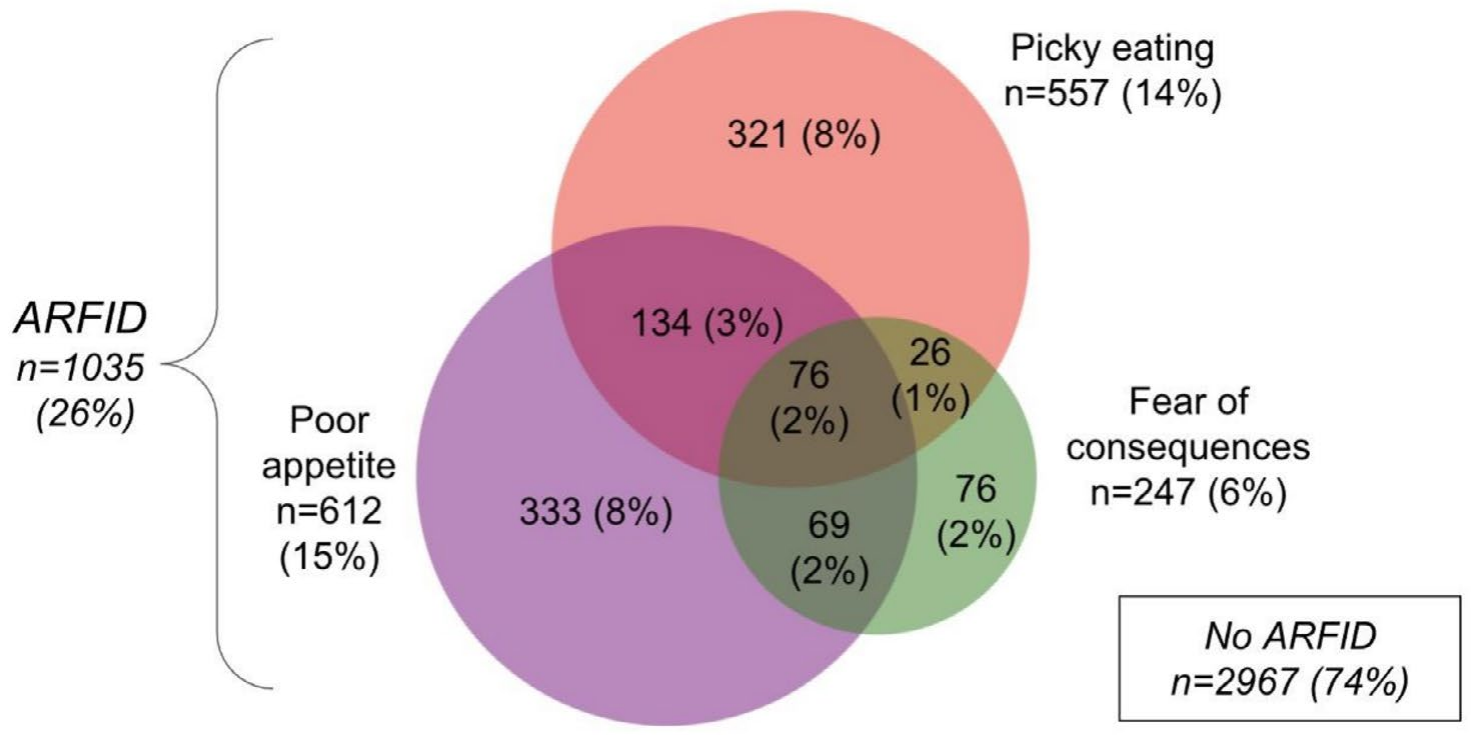
The prevalence of a positive ARFID screen with the adult general population of the UK and USA (*n* = 4002). A positive NIAS screen was based on NIAS‐Picky ≥ 10, NIAS‐Interest ≥ 9, and NIAS‐Fear ≥ 10 (Zickgraf and Ellis 2018; Burton Murray et al. 2021).

In the combined sample, 26% (*n* = 1035 of 4002; Figure 1) screened positive for ARFID symptoms. ARFID was more common in the USA compared to the UK sample (29% vs. 23%, OR 1.36, 95% CI 1.18–1.57, *p* < 0.001).

ARFID symptoms were more common in females than males (29.6% vs. 22.1%, OR 1.48, 95% CI 1.28–1.71). Prevalence was highest in adults aged 18–39 years (31.6%), followed by those aged 40–64 years (25.0%), and lowest in those aged 65 and over (16.1%; *p* < 0.001).

On the NIAS subscales, 15% of the total sample met screening cutoffs by NIAS‐Appetite, 14% by NIAS‐Picky Eating, and 6% by NIAS‐Fear (Figure 1). Furthermore, 18% of participants met criteria for only one ARFID domain, 6% met criteria for two domains, and 2% met criteria for all three domains of ARFID.

On the NIAS subscales, among those with a positive ARFID screen (*n* = 1035), 59% (*n* = 612) met screening cutoffs by NIAS‐Appetite, 54% (*n* = 557) by NIAS‐Picky Eating, and 24% (*n* = 247) by NIAS‐Fear. Furthermore, 71% met screening cutoffs for one domain, 22% for two domains, and 7% for all three domains.

### 3.3 Characteristics and Burden of Individuals With ARFID Symptoms

Individuals with ARFID symptoms were significantly younger than those without ARFID (mean age 42.9 vs. 48.6 years), more commonly female (57.2% vs. 47.5%, OR 1.48 [95% CI 1.28 to 1.71]), and of lower median BMI (25.8 vs. 27.0); all *p* < 0.001. The proportion of individuals with a BMI < 18.5 kg/m^2^ was higher in those with ARFID symptoms than without (7.0% vs. 1.5%, *p* < 0.001). Other BMI category frequencies in ARFID vs. non‐ARFID were: 37% vs. 35% “normal” (18.5–24.9 kg/m^2^), 25.4% vs. 32.3% “overweight” (25–29.9 kg/m^2^), and 30.6% vs. 31.1% “obesity” (≥ 30 kg/m^2^).

On univariate and multivariate analysis (after adjusting for age and gender) those with ARFID symptoms were significantly more likely than unaffected individuals to have higher levels of anxiety, depression, and medium‐to‐high somatic symptoms, including being “bothered a lot” with numerous bodily somatic symptoms (Table 1). They also had a higher prevalence of disorders of gut‐brain interaction across gut anatomical locations, organic gastrointestinal diseases (e.g., inflammatory bowel disease), fibromyalgia and migraines, plus greater healthcare utilization including a history of abdominal surgery, and medication use for gastrointestinal symptoms and mood disturbances. They also experienced significantly reduced mental and physical quality of life.

**TABLE 1:** Characteristics and burden in adults with or without ARFID symptoms.

	No ARFID (*n* = 2967)	ARFID (*n* = 1035)	Unadjusted OR (95% CI)	Adjusted OR^a^ (95% CI)
Anxiety (PHQ‐4 subscale ≥ 3)	620 (20.9%)	445 (43.0%)	2.86 (2.45–3.32)	2.46 (2.10–2.88)
Depression (PHQ‐4 subscale ≥ 3)	607 (20.5%)	425 (41.1%)	2.71 (2.33–3.16)	2.39 (2.05–2.80)
Medium/high somatic symptom reporting (PHQ‐15 ≥ 10)	793 (26.7%)	534 (51.6%)	2.92 (2.52–3.38)	2.51 (2.15–2.92)
“Bothered a lot” somatic symptom reporting (as per PHQ‐15)				
Back pain	551 (18.6%)	334 (32.3%)	2.09 (1.75–2.45)	2.02 (1.72–2.38)
Joint pain	507 (17.1%)	282 (27.2%)	1.82 (1.54–2.15)	1.93 (1.62–2.29)
Headaches	279 (9.4%)	215 (20.8%)	2.52 (2.08–3.07)	2.13 (1.74–2.59)
Chest pain	57 (1.9%)	53 (5.1%)	2.76 (1.88–4.03)	2.39 (1.62–3.53)
Dizziness	105 (3.5%)	90 (8.7%)	2.60 (1.94–3.47)	2.21 (1.64–3.53)
Fainting spells	29 (1.0%)	27 (2.6%)	2.71 (1.60–4.61)	2.26 (1.32–3.87)
Palpitations	124 (4.2%)	96 (9.3%)	2.34 (1.78–3.09)	1.97 (1.49–2.61)
Shortness of breath	141 (4.8%)	122 (11.8%)	2.68 (2.08–3.45)	2.57 (1.98–3.32)
Dyspareunia	75 (2.5%)	55 (5.3%)	2.16 (1.52–3.09)	2.01 (1.40–2.88)
Tiredness	711 (24%)	453 (43.8%)	2.47 (2.13–2.87)	2.14 (1.83–2.49)
Insomnia	645 (21.7%)	423 (40.9%)	2.49 (2.14–2.90)	2.25 (1.93–2.62)
Menstrual cramps	235 (7.9%)	154 (14.9%)	2.03 (1.64–2.52)	1.37 (1.06–1.77)
Abdominal pain	144 (4.9%)	135 (13.0%)	2.94 (2.30–3.76)	2.45 (1.91–3.16)
Bowel disturbances	254 (8.6%)	194 (18.7%)	2.46 (2.01–3.02)	2.23 (1.81–2.74)
Nausea, gas, or indigestion	202 (6.8%)	155 (15.0%)	2.41 (1.93–3.01)	2.11 (1.68–2.65)
Quality of life (PROMIS 10)				
Below average physical QOL	1766 (59.5%)	790 (76.3%)	2.19 (1.87–2.58)	2.14 (1.81–2.52)
Below average mental QOL	1877 (63.3%)	791 (76.4%)	1.88 (1.60–2.21)	1.63 (1.38–1.93)
Disorders of Gut‐Brain Interaction (DGBI)	1114 (37.5%)	590 (57.0%)	2.21 (1.91–2.55)	1.99 (1.72–2.30)
Esophageal DGBI	234 (7.9%)	170 (16.4%)	2.30 (1.86–2.84)	2.20 (1.77–2.73)
Gastroduodenal DGBI	333 (11.2%)	322 (31.1%)	3.57 (3.00–4.25)	3.22 (2.70–3.84)
Functional dyspepsia	208 (7.0%)	267 (25.8%)	4.61 (3.78–5.62)	4.17 (3.41–5.10)
Bowel DGBI	871 (29.4%)	431 (41.6%)	1.72 (1.48–1.99)	1.54 (1.33–1.79)
Irritable bowel syndrome	120 (4.0%)	122 (11.8%)	3.17 (2.44–4.12)	2.69 (2.06–3.52)
Anorectal DGBI	212 (7.1%)	152 (14.7%)	2.24 (1.79–2.79)	2.12 (1.69–2.65)
Other medical problems				
Fibromyalgia	83 (2.8%)	58 (5.6%)	2.06 (1.46–2.91)	2.11 (1.49–3.00)
Migraine	399 (13.4%)	194 (18.7%)	1.49 (1.23–1.79)	1.31 (1.08–1.59)
Diabetes	331 (11.2%)	130 (12.6%)	1.14 (0.92–1.42)	1.53 (1.22–1.92)
Coeliac disease	22 (0.7%)	11 (1.1%)	1.44 (0.70–2.98)	1.40 (0.67–2.93)
Inflammatory bowel disease	33 (1.1%)	25 (2.4%)	2.20 (1.30–3.72)	2.43 (1.42–4.51)
Gastrointestinal cancer	24 (0.8%)	12 (1.2%)	1.44 (0.72–2.89)	2.09 (1.02–4.27)
Abdominal surgery				
Cholecystectomy	230 (7.8%)	99 (9.6%)	1.26 (0.98–1.61)	1.39 (1.07–1.80)
Appendectomy	303 (10.2%)	121 (11.7%)	1.16 (0.93–1.46)	1.33 (1.06–1.67)
Hysterectomy	162 (5.5%)	74 (7.1%)	1.33 (1.00–1.77)	1.49 (1.09–2.02)
Intestinal resection	61 (2.1%)	32 (3.1%)	1.52 (0.99–2.35)	1.85 (1.19–2.89)
Medication use				
Laxatives	219 (7.4%)	205 (19.8%)	3.10 (2.52–3.81)	3.20 (2.59–3.95)
Antidiarrheals	158 (5.3%)	131 (12.7%)	2.58 (2.02–3.29)	2.53 (1.97–3.24)
Antiemetics	144 (4.9%)	160 (15.5%)	3.59 (2.83–4.55)	3.33 (2.61–4.24)
Acid suppressants	722 (24.3%)	381 (36.8%)	1.81 (1.56–2.11)	1.93 (1.65–2.25)
Antispasmodics	217 (7.3%)	154 (14.9%)	2.22 (1.78–2.76)	2.17 (1.73–2.71)
Analgesia	987 (33.3%)	518 (50.0%)	2.01 (1.74–2.32)	2.05 (1.77–2.38)
Anxiolytics	529 (17.8%)	315 (30.4%)	2.02 (1.71–2.37)	1.85 (1.57–2.18)
Antidepressants	551 (18.6%)	321 (31.0%)	1.97 (1.68–2.32)	1.85 (1.57–2.18)
Sedatives	399 (13.4%)	234 (22.6%)	1.88 (1.57–2.25)	1.83 (1.52–2.20)

^a^ Adjusted for age and gender.

Finally, there were significant albeit weak to moderate correlations between individual NIAS subscales—and the total NIAS score—with anxiety, depression, somatic symptoms, and quality of life scores; all *p* < 0.001 (Table 2).

**TABLE 2:** Correlation between severity of individual NIAS subscales, and the total NIAS score, with general health impairment.

	NIAS‐picky eating	NIAS‐Interest	NIAS‐fear	NIAS‐total
Anxiety	*r* = 0.14	*r* = 0.26	*r* = 0.22	*r* = 0.26
Depression	*r* = 0.16	*r* = 0.30	*r* = 0.24	*r* = 0.30
Somatic symptoms	*r* = 0.17	*r* = 0.31	*r* = 0.36	*r* = 0.35
Physical quality of life	*r* = −0.16	*r* = −0.30	*r* = −0.30	*r* = −0.31
Mental quality of life	*r* = −0.14	*r* = −0.24	*r* = −0.19	*r* = −0.24

*Note*: All correlations were significant (*p* < 0.001).

## 4 Discussion

This study demonstrates that one in four adults in the general population of the UK and USA screen positive for ARFID symptoms and experience significantly greater health burdens than unaffected individuals. For example, individuals with a positive ARFID screen were approximately 2.5 times as likely to have elevated scores for anxiety, depression, physical symptom distress and irritable bowel syndrome, and almost four times more likely to have functional dyspepsia.

Interestingly, we found ARFID symptoms were more prevalent in females than males, which contrasts with pediatric literature suggesting ARFID is relatively more male‐predominant in younger populations (Sanchez‐Cerezo et al. 2023). Female predominance in our ARFID symptom group may reflect differences in symptom recognition and reporting between age groups. It may also reflect associations with certain ARFID motivations across the lifespan; for example, initial evidence supports that the fear of aversive consequences presentation has an acute onset (Zickgraf et al. 2019), and thus may be more likely to develop at any age. This is in contrast to the sensory sensitivity presentation which is hypothesized to develop during childhood, emerging from persistent picky eating, with a relatively smaller proportion experiencing onset in later childhood (Breiner et al. 2024). Additionally, disorders of gut‐brain interaction—which had a higher prevalence in our ARFID symptom group—affect approximately 40% of the adult general population and have a female preponderance (Sperber et al. 2021). As has been suggested by others, a subset of individuals with disorders of gut‐brain interaction may develop ARFID in the context of their GI symptoms (Mikhael‐Moussa et al. 2025; Murray et al. 2022; Weeks et al. 2023). Future studies should identify the temporal nature of these associations.

This study has several notable methodological strengths, including its large sample size and homogeneous methodology across the UK and USA. We minimized selection bias by embedding the ARFID screen within a broader general health survey without explicitly mentioning eating disorders. The inclusion of multiple validated questionnaires assessing diverse health outcomes provides a comprehensive assessment of the physical and mental health burden experienced by adults with ARFID, which is generally lacking within the literature.

However, several methodological limitations warrant consideration. First, while the NIAS is a validated screening tool, it was derived from clinical eating disorder populations, and its transferability to the general population remains unconfirmed (Zickgraf and Ellis 2018; Burton Murray et al. 2021). Second, our prevalence estimate of 26% falls at the upper end of the range reported in previous non‐clinical samples (0.8%–28%) (Nicholls‐Clow et al. 2024). This higher estimate likely reflects our use of a screening instrument rather than full diagnostic criteria, consistent with other studies using similar methodology. For example, elevated NIAS scores can result from other eating disorders, gastrointestinal conditions, or normative picky eating rather than ARFID specifically (Burton Murray et al. 2021; Fink et al. 2022). We also did not screen for *DSM‐5* exclusion criteria for ARFID (medical, cultural, religious, or availability‐related food avoidance), potentially leading to misclassification. Therefore, future studies should incorporate systematic screening for other eating disorders (e.g., anorexia nervosa) alongside ARFID‐specific symptom checklists to assess medical and psychosocial impairment, thereby improving diagnostic accuracy (Burton Murray et al. 2021). Nonetheless, it is important to note that the NIAS serves as a preliminary screening tool rather than for diagnostic purposes, and clinical follow‐up remains essential to explore eating behaviors and confirm diagnoses. Early identification through screening can facilitate timely intervention, reduce illness duration and complications, and improve recovery rates. Third, the cross‐sectional design limits causal inference and temporal pattern assessment. Longitudinal studies are needed to understand the natural course of ARFID and its relationship with associated health impairments, particularly disorders of gut‐brain interaction and psychopathology where bidirectional relationships have been demonstrated (Burton‐Murray et al. 2022, 2021). Fourth, gender identity was not captured, which will be important for future studies to understand the prevalence of ARFID among gender identity groups. Fifth, while our sample achieved age and gender representativeness, it was not matched for other potentially relevant factors such as race/ethnicity, education, or socioeconomic status. Moreover, the study was limited to the US and UK—due to funding, investigators' affiliations, and the questionnaires being validated in English (at the time of the study)—thus, future studies in other regions of the world and in other languages are needed. Finally, participants were recruited through Qualtrics’ online service, which may over‐represent certain demographics (e.g., individuals with internet access, higher digital literacy), and the survey company was unable to capture the denominator to provide a responder rate. This recruitment methodology could potentially impact the generalizability of our prevalence estimates.

The substantial multi‐morbidity and healthcare utilization associated with positive ARFID screening supports implementing multidisciplinary care approaches (Fisher et al. 2023). Given the strong associations with psychological co‐morbidity and gastrointestinal disorders, routine ARFID screening in such clinics is advisable. This should be considered regardless of BMI, as ARFID affects individuals of any body size. Within GI clinics, physicians should recognize that standard dietary recommendations for digestive conditions could inadvertently exacerbate ARFID symptoms. Evidence from disorders of gut‐brain interaction (e.g., irritable bowel syndrome) suggests that multi‐integrated approaches comprising gastroenterologists, dietitians, and psychologists are more effective than single‐specialty care (Basnayake et al. 2020). While outside the scope of this article, similar multidisciplinary care approaches should be adopted for adults with ARFID, with recent research showing promise (Hellner et al. 2025).

In conclusion, these findings highlight ARFID symptoms are common in the general adult population and associated with a substantial health impact. Enhanced clinical awareness, increased service provision, and expanded research into this poorly understood condition will lead to improved outcomes for individuals affected by ARFID.

## Author Contributions

**Grace Brownlow:** writing – original draft, writing – review and editing, formal analysis, data curation, project administration, investigation. **Rosie Flack:** writing – original draft, writing – review and editing, formal analysis, project administration, data curation, investigation. **Helen Burton‐Murray:** writing – original draft, writing – review and editing, formal analysis. **Olafur Palsson:** conceptualization, investigation, writing – review and editing, writing – original draft, methodology, validation, formal analysis. **Imran Aziz:** conceptualization, investigation, funding acquisition, writing – original draft, methodology, validation, visualization, writing – review and editing, formal analysis, supervision.

## Ethics Statement

Before data collection started, the study was reviewed by the Institutional Review Board (IRB) at the University of North Carolina (Chapel Hill, NC, USA), and the University of Sheffield (Sheffield, UK). It was deemed IRB exempt because all study participants were anonymous to the investigators. All authors had access to the study data and reviewed and approved the final manuscript.

## Conflicts of Interest

G.B., R.F., O.P., and I.A. declare no relevant conflicts of interest. H.B.‐M. receives royalties from Oxford University Press for her book *Cognitive Behavioral Therapy for Rumination Syndrome* and from Cambridge University Press for her forthcoming book on functional bowel disorders.

## Supporting information


**Data S1:** Supporting Information.

## Data Availability

The data that support the findings of this study are available from the corresponding author upon reasonable request.
