# Supplementary material for: The Prevalence and Burden of Avoidant/Restrictive Food Intake Disorder (ARFID) Symptoms in the Adult General Population of the UK and USA
Source: Int J Eat Disord. 2025 Oct 29;59(2):362–70. doi: 10.1002/eat.24588 (PMC12884231; doi:10.1002/eat.24588)
Supplement: Supplementary file 1 — Data S1: Supporting Information. [file EAT-59-362-s001.doc]

**SUPPLEMENTARY**

**Table :** Participant demographics.

|  | Overall (n=4002) | UK (n=2002) | USA (n=2000) |
| --- | --- | --- | --- |
| Female gender^*^, n (%) | 2000 (50.0%) | 1000 (50.0%) | 1000 (50.0%) |
| **Age categories, n (%)** |  |  |  |
| 18-39 yrs | 1596 (39.9%) | 798 (39.9%) | 798 (39.9%) |
| 40-64 yrs | 1606 (40.1%) | 805 (40.2%) | 801 (40.1%) |
| 65+ yrs | 800 (20.0%) | 399 (19.9%) | 401 (20.1%) |
| **Ethnicity, n (%)****  Asian  Black/African-American  Hispanic  White/Caucasian  Other or Mixed ethnicity | n=3972  171 (4.3%)  312 (7.9%)  115 (2.9%)  3246 (81.7%)  128 (3.2%) | n=1985  114 (5.7%)  80 (4.0%)  0 (0.0%)  1728 (87.1%)  63 (3.2%) | n=1987  57 (2.9%)  232 (11.7%)  115 (5.8%)  1518 (76.4%)  65 (3.3%) |

^*^ Self-report question of “what is your gender?” with response options of “male” and “female”.

^**^ Ethnicity was not available in all participants
